# Supplementary material for: The interactome of intact mitochondria by cross-linking mass spectrometry provides evidence for coexisting respiratory supercomplexes
Source: Mol Cell Proteomics. 2017 Dec 8;17(2):216–32. doi: 10.1074/mcp.RA117.000470 (PMC5795388; doi:10.1074/mcp.RA117.000470)
Supplement: Supplemental Data [file supp_RA117.000470_133922_0_supp_23978_fzffwf.zip › spectra_annotation/mito_DR_spectra_annotation/322-1-10-1-15-1.pdf]

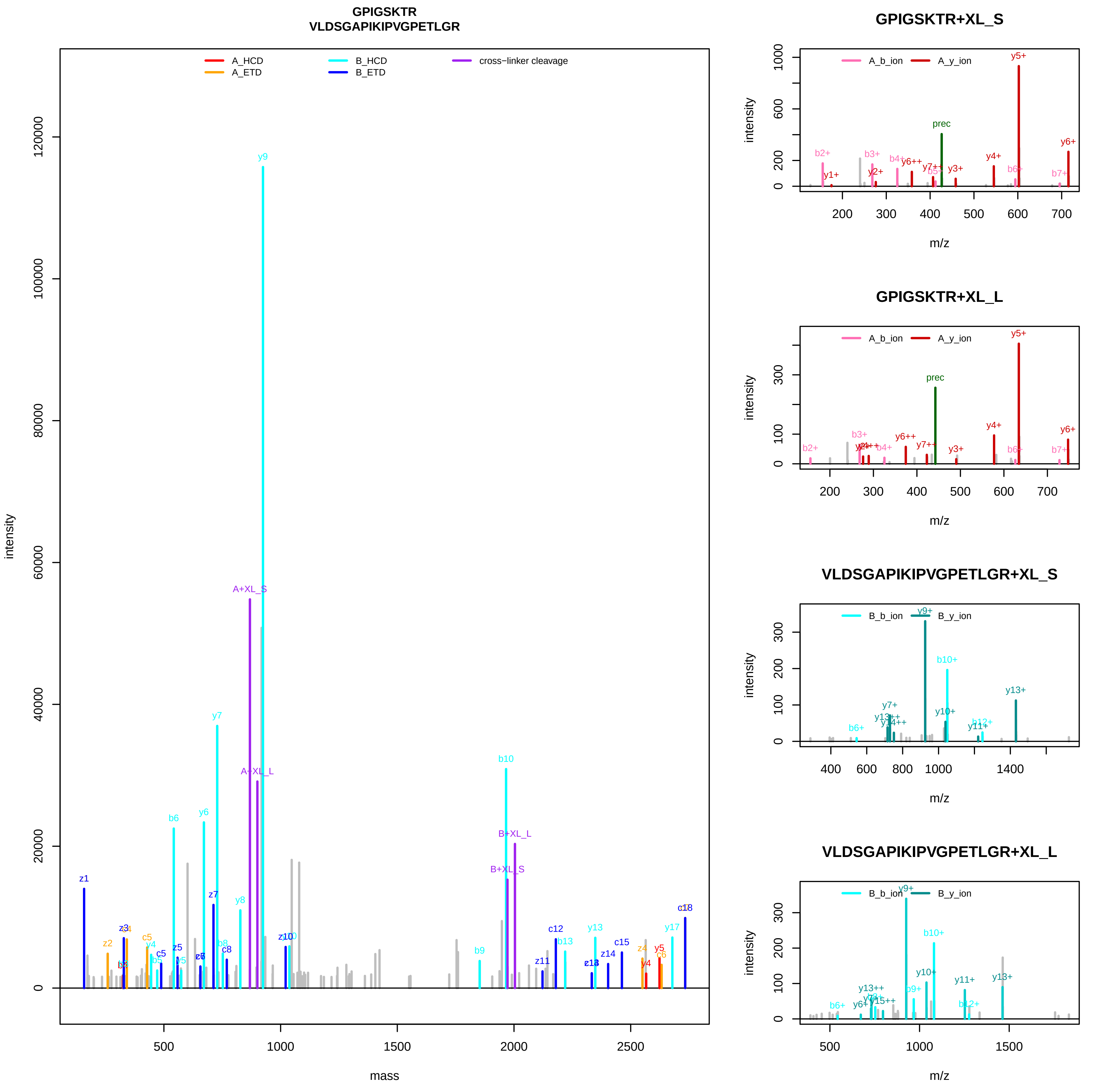
GPIGSKTR
VLDSGAPIKIPVGPETLGR
A_HCD
A_ETD
B_HCD
B_ETD
cross−linker cleavage
intensity
mass
y9
A+XL_S
A+XL_L
y7
b6
y6
b10
B+XL_L
B+XL_S
z1
z7
y8
z3
z2
c5
y4
z5
c8
z10
c12
b13
y13
c15
z14
z11
y17
c18
y5
c6
z4
y4
b9
0
20000
40000
60000
80000
100000
120000
500
1000
1500
2000
2500
GPIGSKTR+XL_S
A_b_ion
A_y_ion
y5+
prec
y6+
b2+
y1+
b3+
y2+
b4+
y6++
y7++
b5+
y3+
y4+
b6+
b7+
m/z
GPIGSKTR+XL_L
VLDSGAPIKIPVGPETLGR+XL_S
B_b_ion
B_y_ion
y9+
b10+
y13+
y7+
y13++
y14++
b6+
y10+
y11+
b12+
VLDSGAPIKIPVGPETLGR+XL_L
y15++
b9+
